# Malarial Hemozoin Is a Nalp3 Inflammasome Activating Danger Signal

**DOI:** 10.1371/journal.pone.0006510

**Published:** 2009-08-04

**Authors:** Catherine Dostert, Greta Guarda, Jackeline F. Romero, Philippe Menu, Olaf Gross, Aubry Tardivel, Mario-Luca Suva, Jean-Christophe Stehle, Manfred Kopf, Ivan Stamenkovic, Giampietro Corradin, Jurg Tschopp

**Affiliations:** 1 Department of Biochemistry, University of Lausanne, Epalinges, Switzerland; 2 Division of Experimental Pathology, Institute of Pathology, CHUV, Lausanne, Switzerland; 3 Institut Integrative Biologie, ETH, Zürich, Switzerland; New York University School of Medicine, United States of America

## Abstract

**Background:**

Characteristic symptoms of malaria include recurrent fever attacks and neurodegeneration, signs that are also found in patients with a hyperactive Nalp3 inflammasome. *Plasmodium* species produce a crystal called hemozoin that is generated by detoxification of heme after hemoglobin degradation in infected red blood cells. Thus, we hypothesized that hemozoin could activate the Nalp3 inflammasome, due to its particulate nature reminiscent of other inflammasome-activating agents.

**Methodology/Principal Findings:**

We found that hemozoin acts as a proinflammatory danger signal that activates the Nalp3 inflammasome, causing the release of IL-1β. Similar to other Nalp3-activating particles, hemozoin activity is blocked by inhibiting phagocytosis, K^+^ efflux and NADPH oxidase. *In vivo*, intraperitoneal injection of hemozoin results in acute peritonitis, which is impaired in Nalp3-, caspase-1- and IL-1R-deficient mice. Likewise, the pathogenesis of cerebral malaria is dampened in Nalp3-deficient mice infected with *Plasmodium berghei* sporozoites, while parasitemia remains unchanged.

**Significance/Conclusions:**

The potent pro-inflammatory effect of hemozoin through inflammasome activation may possibly be implicated in plasmodium-associated pathologies such as cerebral malaria.

## Introduction

Malaria infects 300–500 million and kills more than one million children annually. The causative agents of malaria, *Plasmodium* species, go through a complex life cycle, involving both a mosquito vector and the human host [1]. In infected individuals the parasite first enters the clinically silent “liver stage” followed by a “blood stage”, which is characterized by cyclic red blood cell lysis resulting in fever peaks, chills and anemia [2]. In less than 1–2% of cases, severe malaria can evolve to its most lethal form, cerebral malaria (CM). *Plasmodium* infection elicits in its host an immune response that is characterized mostly by IFNγ producing T cells and antibodies directed against infected red blood cells [3], [4]. However, the immune response is also critically involved in the pathogenesis of severe malaria, largely through the overproduction of pro-inflammatory cytokines [5]. Concerning innate immunity, several conserved molecular structures of *Plasmodium* have been proposed to act as pathogen-associated molecular patterns (PAMPs) and are activating Toll-like receptors (TLRs) on macrophages and dendritic cells (DCs), such as glycosylphosphatidylinositol (GPI), which is a TLR2 ligand [6].

Hemozoin is a heme crystal, which is formed by the parasite in order to detoxify free heme resulting from hemoglobin digestion in the infected red blood cells [7], [8]. *Plasmodium* spp are able to form insoluble hemozoin crystals in order to protect themselves from oxidative damage resulting from the presence of free heme. During red blood cell lysis, hemozoin is released into the blood stream together with the parasite and cellular debris. Purified hemozoin from *P. falciparum*, as well as synthetic hemozoin, can activate macrophages and DCs to produce pro-inflammatory cytokines and chemokines [9], [10]. However, there are conflicting reports on the immunomodulatory capacities of hemozoin crystals *per se*. Hemozoin was reported to activate TLR9 signalling, and according to that TLR9- and MyD88-deficient mice were shown to be less susceptible to CM [10], [11]. Yet these results were recently questioned [12], as signalling triggered by *P. falciparum*-derived hemozoin was shown to be dependent on the presence of malarial DNA complexed to hemozoin, hence explaining the reported implication of TLR9 [13].

The pro-inflammatory cytokines IL-1β and IL-18 are produced by cleavage of the inactive proIL-1β and proIL-18 precursors by caspase-1. Caspase-1 is activated within a large multi-protein complex, termed the inflammasome [14], which is triggered by several danger-, stress- and/or infection-associated signals leading to caspase-1 cleavage and activation. The Nalp3 inflammasome, composed of the NLR protein Nalp3, the adaptor ASC and caspase-1, has been shown to be implicated in the production of mature IL-1β. It is now generally accepted that activation and release of IL-1β requires two distinct signals: the first signal leads to the transcriptional upregulation and synthesis of proIL-1β and other components necessary for inflammasome function, such as Nalp3 itself; the second signal leads to Nalp3 inflammasome complex formation, caspase-1 activation and IL-1β cleavage. This signal is constituted of an ever growing number of different stimuli such as bacterial and viral PAMPs [15], stress-associated danger signals such as ATP or MSU, and other particulate stimuli such as asbestos, silica, alum and β-amyloid [16], while the actual mechanism by which Nalp3 activation leads to caspase-1 cleavage remains unknown. In this study we investigated whether hemozoin could act as a Nalp3 inflammasome activating danger signal leading to IL-1β production.

## Results

### Hemozoin induces IL-1β secretion in myeloid cells

**Figure 1:**
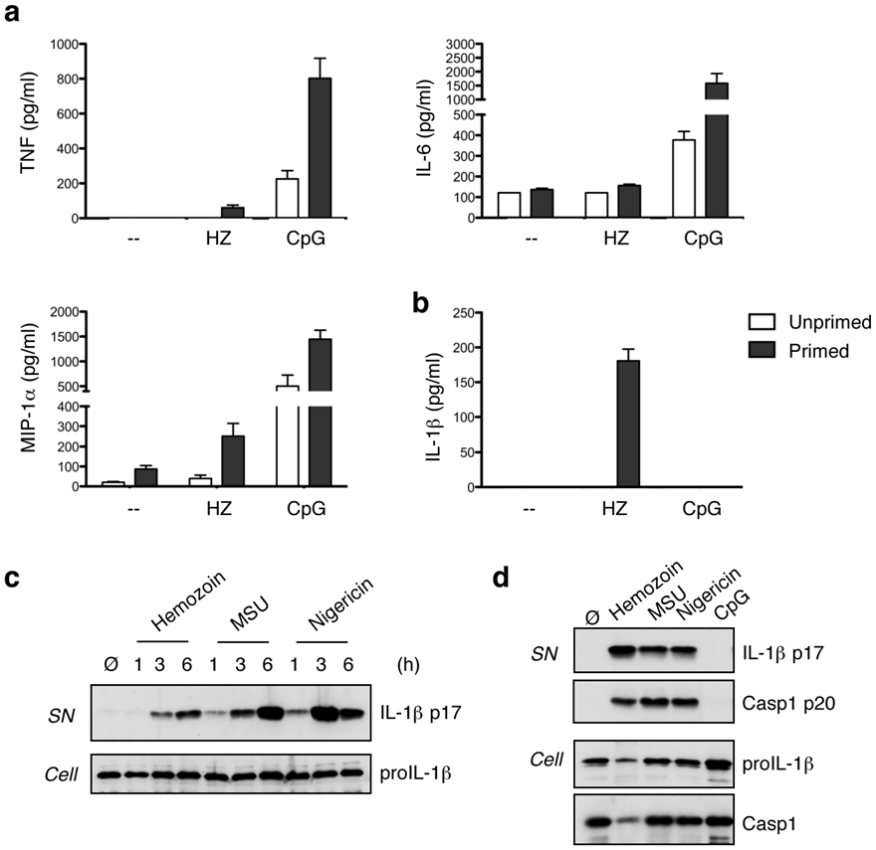
Hemozoin activates IL-1β secretion in murine and human macrophages and dendritic cells. (a,b) Bone marrow derived macrophages (BMDMs) were stimulated for 6 h with 150 µg/ml hemozoin and 2.5 µg/ml CpG. Cytokines and chemokines were measured by CBA. (c) THP1 cells were stimulated for 1, 3 or 6 h with hemozoin, MSU or Nigericin and analysed by western blot. (d) Bone marrow derived dendritic cells (BMDCs) were stimulated for 6 h with hemozoin, MSU, Nigericin or CpG. Cell extracts and supernatants were analysed by western blot. Data shown are representative of three independent experiments.

In an attempt to determine more precisely the immunostimulatory capacities of hemozoin crystals, we produced synthetic hemozoin (also called β-hematin), which is free of malarial DNA. Bone marrow-derived macrophages (BMDMs) stimulated with hemozoin produced relatively low levels of TNFα, IL-6 and MIP-1α after 6 hours stimulation, as compared to stimulation with TLR9-activating CpG **(** **Figure 1a** **)**. On the other hand, BMDMs stimulated with hemozoin were able to robustly secrete IL-1β and IL-18 when primed with LPS **(** **Figure 1b** **and Figure S1a)**. In order to rule out any species- or cell type-specific effects, we tested the ability of hemozoin to induce IL-1β production in the human macrophage-like cell line THP1 **(** **Figure 1c** **)**, as well as in murine bone marrow-derived dendritic cells (BMDCs) **(** **Figure 1d** **)**. IL-1β secretion was observed in both cell types in a time- and dose-dependent manner **(** **Figure 1c** **and Figure S1b)**.

### Hemozoin-induced IL-1β secretion is Nalp3 inflammasome-dependent

**Figure 2:**
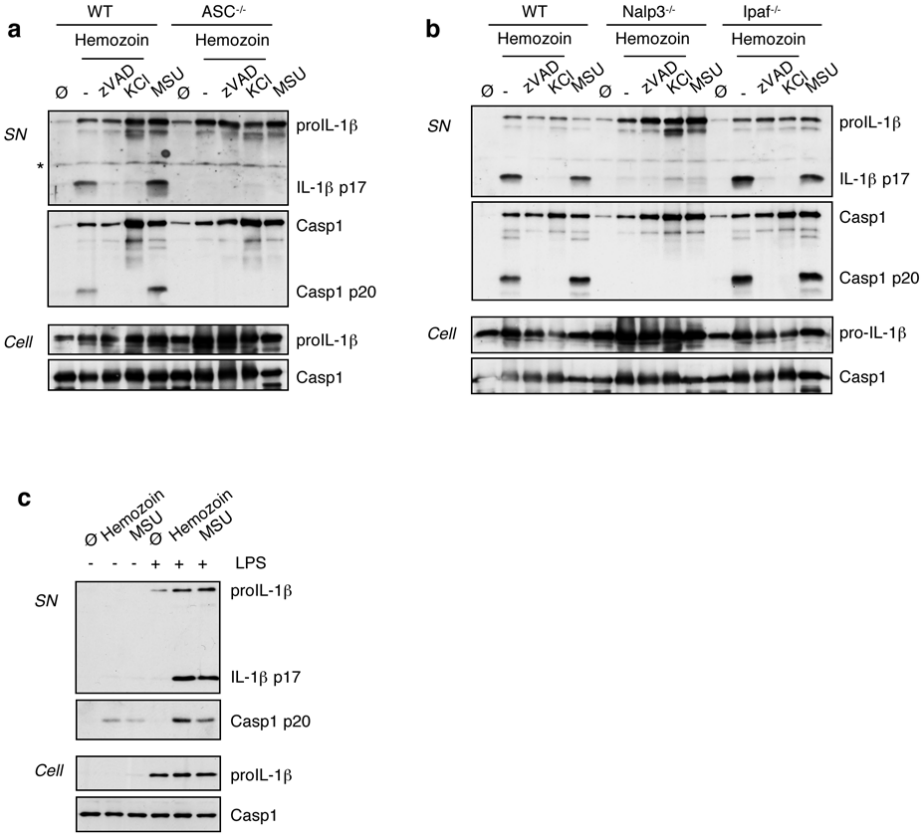
Hemozoin induced IL-1β secretion is NALP3 inflammasome-dependent. (a,b) BMDMs from wild-type (WT), ASC-, Nalp3- or Ipaf-deficient mice were stimulated for 6 h with hemozoin (150 µg/ml) or MSU (150 µg/ml ) in the presence of 20 µM z-VAD or 130 mM KCl where indicated (* nonspecific band). (c) BMDMs were primed or not with LPS before stimulation with hemozoin or MSU. Data shown are representative of three independent experiments.

**Figure 3:**
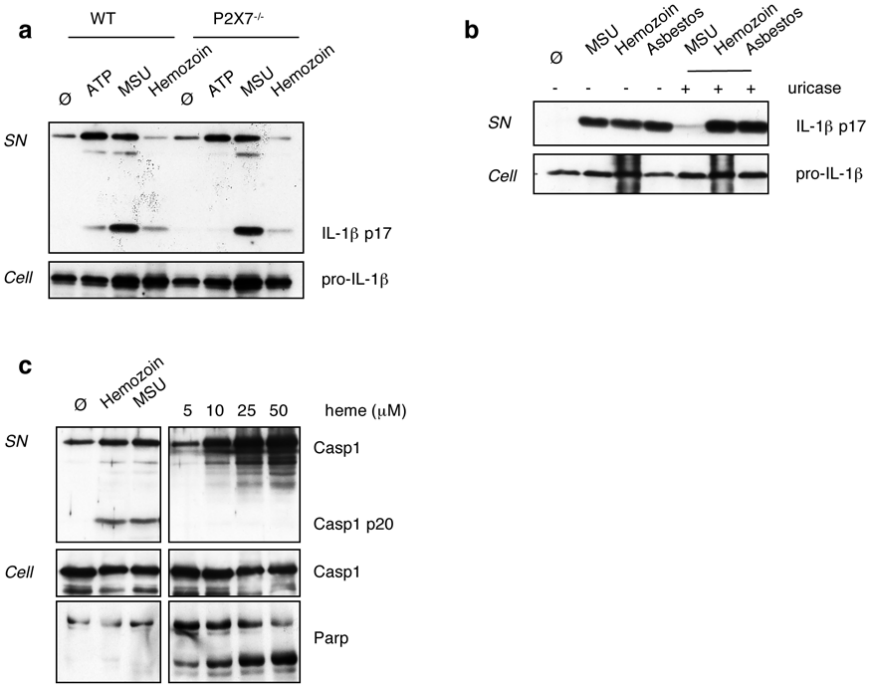
Hemozoin induced IL-1β production is independent from P2X7 activation. (a) BMDMs were stimulated for 45 min with 5 mM ATP, or for 6 h with 100 µg/ml hemozoin and 100 µg/ml MSU. (b) Uricase treatment (0,1 U/ml) of THP1 cells does not affect IL-1β production by hemozoin. Cells were stimulated with 150 µg/ml hemozoin, 150 µg/ml asbestos and 50 µg/ml MSU. (c) Heme does not activate caspase-1 cleavage in BMDMs as compared to hemozoin (150 µg/ml) or MSU (150 µg/ml), but leads to PARP cleavage, indicating its toxicity. Cell supernatants and extracts were analysed by Western blot.

**Figure 4:**
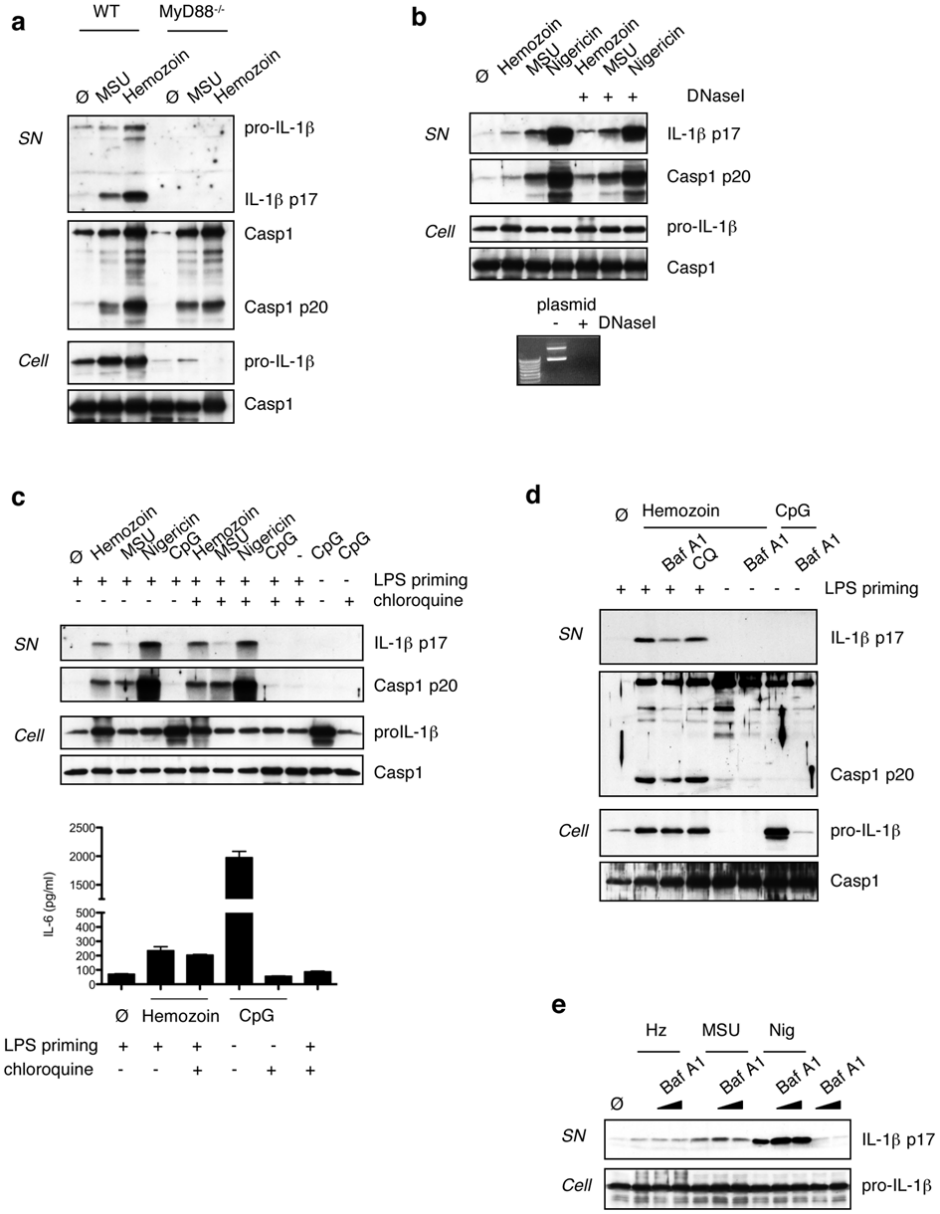
Hemozoin induced IL-1β production is independent from MyD88-mediated signaling pathways. (a) Caspase-1 activation can still be observed in MyD88-deficient BMDMs, albeit to a lesser extent. (b) BMDMs were stimulated with hemozoin, MSU and Nigericin in the presence or absence of DNaseI (100 U/ml). Cells were stimulated for 6 h with 150 µg/ml hemozoin, 150 µg/ml MSU and 1.34 µM Nigericin. Cell supernatants and extracts were analysed by Western blot. (c) BMDMs were stimulated with hemozoin, MSU, Nigericin or CpG (2,5 µg/ml) in the presence or absence of 10 µM chloroquine. (d,e) BMDMs (d) and THP1 cells (e) were stimulated with hemozoin, MSU, nigericin and CpG in the presence or absence of bafilomycinA1 (250 nM in (d) and 50 and 250 nM in (e)). IL-1β secretion and caspase-1 cleavage were analysed by western blot; IL-6 was analysed by CBA.

The Nalp3 inflammasome is implicated in the production of mature IL-1β and IL-18 in response to different signals among which there are several particulate stimuli, such as MSU, alum or asbestos [16]. The precise mechanism of Nalp3 inflammasome activation is still poorly understood. We found that hemozoin-induced IL-1β secretion was blocked by the pan-caspase inhibitor z-VAD, where cleaved caspase-1 was no longer observed **(** **Figure 2a** **)** and no IL-1β secretion could be observed in caspase-1-deficient macrophages stimulated with hemozoin or any inflammasome activator tested **(Figure S1d)**. In addition to caspase-1, the inflammasome components ASC and Nalp3 were required for IL-1β production in response to hemozoin **(** **Figure 2a, 2b** **and Figure S1c)**. In contrast, Ipaf, another NLR protein shown to form an inflammasome responsible of IL-1β production in response to bacteria such as *Salmonella* [17], was not essential for hemozoin-induced IL-1β secretion **(** **Figure 2b** **)**. In unprimed cells hemozoin was still able to induce caspase-1 cleavage although to a lesser extent, clearly showing its potent inflammasome-activating capacities **(** **Figure 2c** **)**. Hemozoin-induced IL-1β production was not mediated by ATP released from dying cells as a consequence of hemozoin toxicity, as shown by the use of P2X7-deficient BMDMs **(** **Figure 3a** **)**. Likewise, uric acid crystals, that can act as an endogenous danger signal produced upon cellular stress, are not involved in hemozoin-mediated signal transmission, as inflammasome activation was not altered in the presence of uricase **(** **Figure 3b** **)**. In agreement with previous results, we found that heme, the precursor of hemozoin, does not activate caspase-1 but is toxic, as seen by PARP cleavage **(** **Figure 3c** **)**. Importantly, hemozoin treatment did not result in PARP cleavage, indicating that the hemozoin preparation used is not toxic and free of contaminating heme. In order to exclude any implication of DNA-mediated TLR9 signalling in IL-1β production by hemozoin as proposed [13], we used MyD88-deficient macrophages where caspase-1 cleavage was still observed, although there was no IL-1β secreted as expected **(** **Figure 4a** **)**. In addition, treatment of hemozoin with DNaseI had no effect on IL-1β production **(** **Figure 4b** **)**. Chloroquine is a well-known antimalarial drug, but its exact mechanism of action in malaria treatment is still unknown. It has been shown that chloroquine can interfere with the hemozoin crystallization process in infected red blood cells [18]. Chloroquine is also known to block endosomal acidification, resulting in improper TLR9 signalling [19]. Treatment with chloroquine prior to BMDM stimulation with hemozoin had no effect on IL-1β production by hemozoin, MSU or nigericin at any of the different chloroquine concentrations tested **(** **Figure 4c** **and Figure S2)**. Nevertheless, chloroquine inhibited CpG-dependent IL-6 production, whereas hemozoin-induced IL-6 and MIP-1α production was independent of TLR9 **(** **Figure 4c** **and data not shown)**. We also tested the effect of bafilomycin A1 on inflammasome activation in response to hemozoin. Bafilomycin blocks the vacuolar H^+^ ATPase system necessary for lysosomal acidification and has been shown to inhibit inflammasome activation in response to silica [20]. We could not observe this effect on inflammasome activation by hemozoin, MSU or nigericin both in BMDMs and in THP1 cells **(** **Figure 4d,e** **)**. However, bafilomycin could strongly reduce proIL1β induction in response to stimulation with CpG **(** **Figure 4d** **)**.

**Figure 5:**
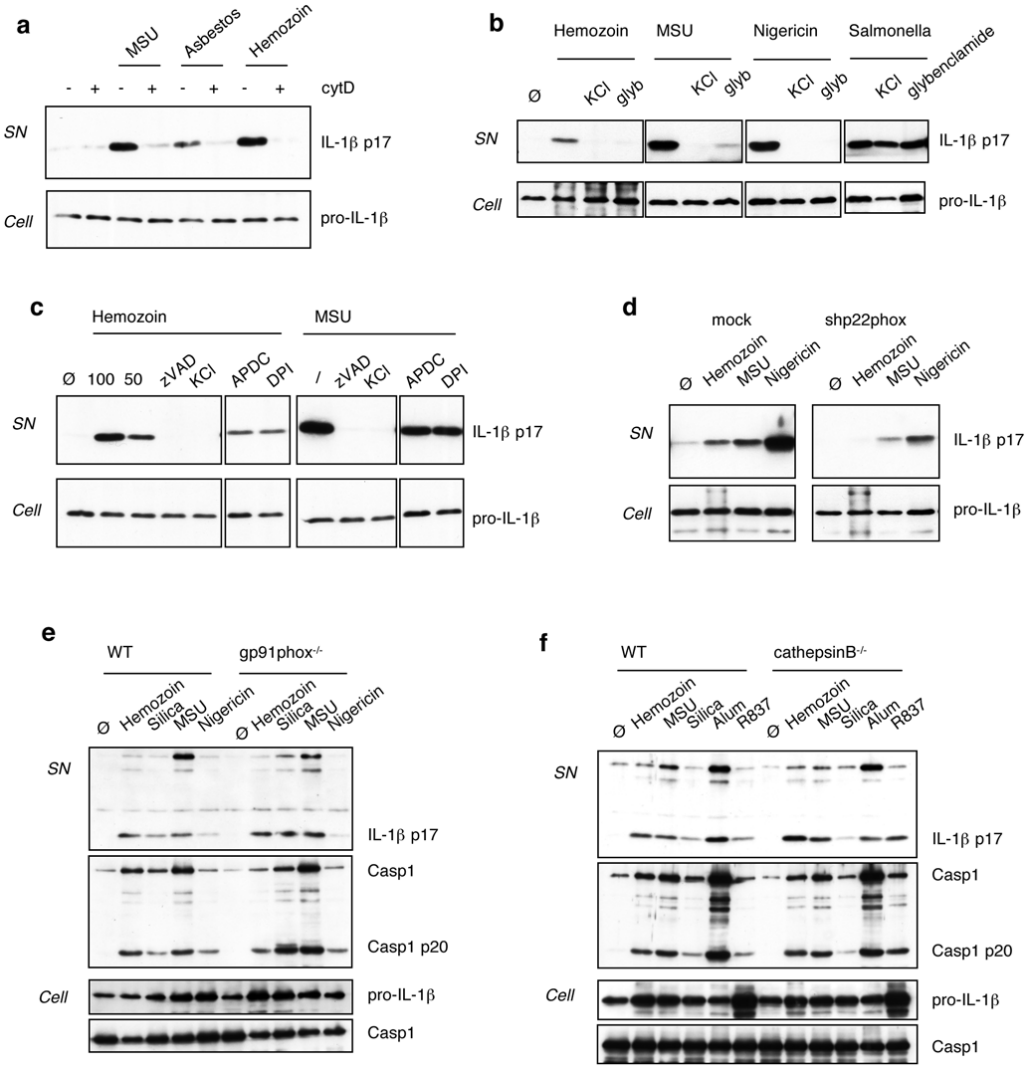
Phagocytosis, K^+^ efflux and activation of a NADPH oxidase are essential for hemozoin-mediated inflammasome activation. (a) Crystal phagocytosis is essential for hemozoin induced IL-1β production as evidenced by the use of cytochalasin D (2 µM) to block the actin cytoskeleton. (b) Hemozoin induced IL-1β production can be reduced by blocking the K^+^ efflux from the cells by adding high extracellular K^+^ concentration to the culture medium (130 mM KCl) or by using glybenclamide, an ATP-sensitive potassium channel inhibitor (50 µM). (c) Hemozoin induced IL-1β production can be reduced by the use of ROS inhibitors, such as APDC (50 µM) and DPI (20 µM). (d) NADPH oxidase subunit p22phox is essential for inflammasome activation by hemozoin. (e) gp91phox(NOX2)-deficient BMDMs were stimulated with different crystals and nigericin for 6 h. (f) Cathepsin B-deficient BMDMs were stimulated with the indicated inflammasome activators for 6 h. THP1 cells were stimulated for 6 h with 150 µg/ml hemozoin, 100 µg/ml MSU, 100 µg/ml asbestos and 1,34 µM Nigericin. Salmonella were added to the cells at an MOI of 10. BMDMs were stimulated with 150 µg/ml hemozoin, 150 µg/ml MSU, 250 µg/ml silica, 150 µg/ml alum, 15 µg/ml R837 and 1,34 µM Nigericin Cell supernatants and extracts were analysed by Western blot.

Phagocytosis of hemozoin crystals is necessary for Nalp3 inflammasome activation as shown by the ability of cytochalasin D to suppress IL-1β production **(** **Figure 5a** **)**. Similar to Nalp3 inflammasome activation by other particulate stimuli such as MSU or asbestos, hemozoin-induced IL-1β production was inhibited by blocking the K^+^ efflux from the cell by using the ATP-sensitive potassium channel inhibitor glybenclamide **(** **Figure 2a,b** **and** **Figure 5b** **)**. Also, generation of ROS is implicated in hemozoin-induced IL-1β production, as demonstrated by experiments using the NADPH oxidase inhibitor DPI or cells with p22phox levels that are reduced by RNAi **(** **Figure 5c,d** **)**. The exact source of ROS is still unclear, since the gp91phox (NOX2) subunit of the complex does not seem to be required for inflammasome activation (**Figure 5e** and [20] ). This observation suggests the implication of one of the several other NOX isoforms in this process. An additional mechanism of inflammasome activation based on lysosomal destabilisation and release of cathepsin B in response to crystal phagocytosis was recently proposed [20]. We took advantage of cathepsin B-deficient BMDMs to test this hypothesis, however no differences in IL-1β secretion and caspase-1 cleavage in response to several inflammasome activators, such as hemozoin, MSU or alum were observed (**Figure 5f**). Similar results were observed in bone-marrow derived dendritic cells (**data not shown**).

### Hemozoin has pro-inflammatory properties *in vivo*

**Figure 6:**
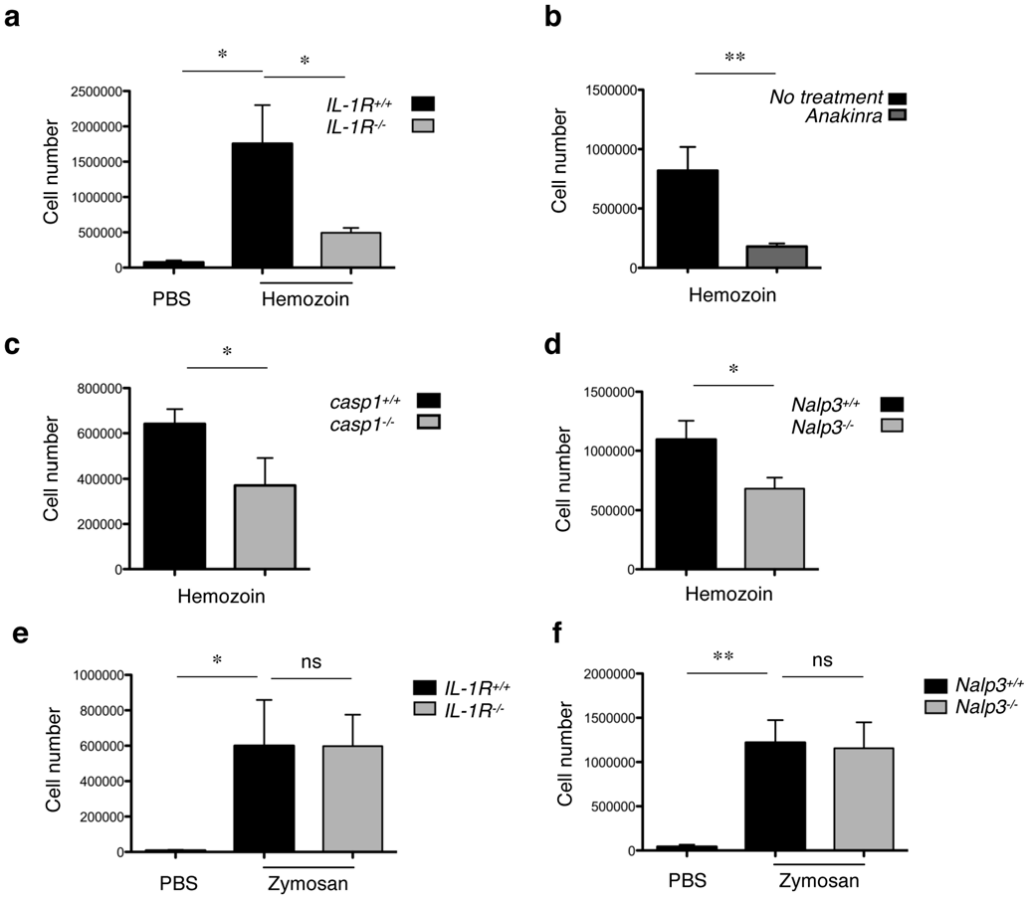
Role of the inflammasome in a mouse model of hemozoin-induced peritonitis. (a-f) The indicated WT or mutant mice were injected intraperitoneally with 0.25 mg of hemozoin, 0.2 mg of zymosan or PBS as a control. Neutrophil influx (CD11b^+^ Ly-6C^+^ Ly-6G^high^ F4/80^−^) was quantified 6 h later by FACS (values are mean±s.e.m and *n* = 4–6 mice per group). Differences between groups were calculated using the unpaired t test and were considered significant (*) when p≤0.05. Data shown are representative of three independent experiments.

In order to determine the pro-inflammatory and danger signal capacities of hemozoin *in vivo*, we used a well-established peritonitis model where intraperitoneal injection of inflammasome activators results in neutrophil influx to the peritoneal cavity [20], [21]. Hemozoin elicited a considerable increase in the recruitment of neutrophils 6 h after injection compared to PBS in both mice on a C57BL/6 background (Nalp3^+/+^) and on a BALB/c background (IL-1R^+/+^) **(** **Figure 6** **)**. When hemozoin was injected in mice deficient in IL-1R, neutrophil influx was markedly impaired, implicating a role for IL-1 signalling in attracting neutrophils to the peritoneal cavity **(** **Figure 6a** **)**. In keeping with this, the IL-1 antagonist, IL-1Ra (Anakinra) also efficiently blocked neutrophil recruitment **(** **Figure 6b** **)**. Importantly, reduced neutrophil influx was also observed in caspase-1-deficient mice and Nalp3-deficient mice **(** **Figure 6c and d** **)**, as well as in ASC-deficient mice **(Figure S3a)**. In contrast, zymosan-induced neutrophil influx was not affected by IL-1R- or Nalp3-deficiency, indicating that Nalp3-deficient mice do not have a general defect in neutrophil recruitment **(** **Figure 6e and f** **)**. The reduction in neutrophil recruitment was strongest in IL-1R-deficient and Anakinra-treated mice, suggesting that IL-1α or other signalling pathways participate in hemozoin-induced neutrophil recruitment. Chloroquine treatment had no significant effect, suggesting that TLR9 is not involved in attracting neutrophils to the peritoneal cavity in response to hemozoin **(Figure S3b)**.

### Nalp3 is implicated in the development of cerebral malaria

**Figure 7:**
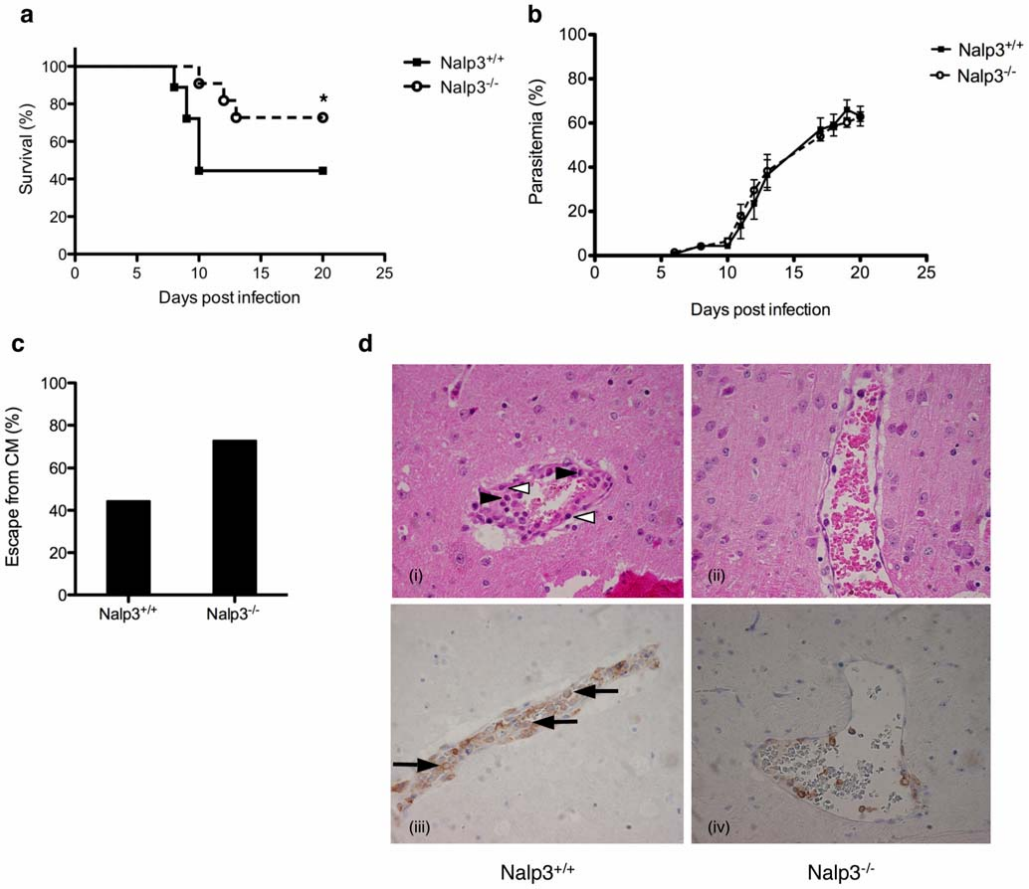
Role of Nalp3 in a mouse model of cerebral malaria. (a-d) Nalp3-deficient mice have increased resistance to cerebral malaria (CM). (a, b) Survival of Nalp3^+/+^ (solid line) and Nalp3^−/−^ (dotted line) mice was monitored daily (*n* = 18–22 for each group, (*) log-rank p = 0.0263) and parasitemia was assessed by blood smears (mean parasitemia±s.e.m., *n* = 6 for each group, N3^+/+^ = black square, N3^−/−^ = open circles). (c) Nalp3^−/−^mice are protected from CM compared to Nalp3^+/+^ mice (*n* = 15 for each group). (d) Histological analysis of brain sections of Nalp3^+/+^ (panel i and iii) and Nalp3^−/−^ (panel ii and iv) mice 9 days after *Plasmodium* infection. Haematoxylin & Eosin staining is shown in panels i and ii, CD45 staining is shown in panels iii and iv. Infected Nalp3^+/+^ mice brains show pathological endothelial cells (open triangles) and lymphomonocytic infiltrates (closed triangles) whereas in the brains from infected Nalp3-deficient mice these pathological signs are markedly reduced (panels i and ii).

Based on the observed inflammasome-activating capacity of hemozoin *in vitro*, we investigated the potential role for the inflammasome in malaria. To this end we used *Plasmodium berghei* ANKA (PbA) infection in mice as a model for cerebral malaria (CM) [22]. Mice were injected intravenously with a low number of PbA sporozoites, which first migrate to the liver where they evolve to form merozoites that are released during the clinically silent liver stage. The merozoites eventually invade erythrocytes leading to their cyclic rupture and re-infection. Mice on a C57Bl/6 background are susceptible for developing the neurological signs of CM and usually die 7–12 days post infection. When wild type (Nalp3^+/+^) and Nalp3-deficient mice were infected with 10 PbA sporozoites, a consistent proportion of Nalp3^+/+^ control mice died from CM after 7–12 days, whereas the Nalp3-deficient mice resisted better to CM development **(** **Figure 7a** **)**. Mice from both groups were similarly infected with PbA, as parasitemia, corresponding to the percentage of infected red blood cells (iRBC), could be monitored for all the mice **(** **Figure 7b** **)**. Increasing parasitemia was observed in both the Nalp3^+/+^ and the Nalp3-deficient mice that resisted CM, and mice were killed three weeks after infection when their iRBC number exceeded 80% and the mice developed hyperparasitemia-induced anaemia, a condition unrelated to CM. Overall, 73% of Nalp3^−/−^ mice were resistant to CM, as compared to only 44% of Nalp3^+/+^ mice **(** **Figure 7c** **)**. We examined immunopathological changes in the brains of Nalp3^+/+^ and Nalp3^−/−^ mice 9 days after infection. Nalp3^+/+^ mice showed typical vascular occlusion with parasitized erythrocytes as well as lymphomonocytic infiltrates and microvascular destruction including pathological endothelial cells **(** **Figure 7d** (i)**)**. In contrast, very limited characteristic pathological changes were detected in Nalp3-deficient mice, such as moderate leukocyte infiltration and damaged endothelial cells, suggesting that Nalp3-dependent immune responses may play a role in the brain pathogenesis of CM **(** **Figure 7d** (ii)**)**. CD45 staining also shows infiltration of leukocytes in the brains of *Plasmodium* infected Nalp3^+/+^ mice, whereas Nalp3^−/−^ mice show reduced CD45 staining **(** **Figure 7d** (iii and iv)**)**.

## Discussion

A better comprehension of the molecular mechanisms leading to malaria is instrumental for the development of novel and more efficient anti-malarial drugs. It is generally accepted that in addition to parasite-associated virulence factors, an exacerbated host inflammatory response to parasite infection resulting in collateral damage, contributes to cerebral malaria [4]. TLRs are thought to play a central role in this response, since they are not only able to detect parasite-specific molecular patterns, but also host-derived hemozoin in a complex with plasmodial DNA [10], [13]. Here we show that in addition to TLRs, hemozoin activates the Nalp3 inflammasome, thus possibly explaining its potent proinflammatory activity.

A plethora of Nalp3 inflammasome-activating substances have been identified. A minority of them are PAMPs of bacterial or viral origin, while most of them are host-derived substances or particles released into the environment (DAMPs). The most active known endogenous danger signals are ATP and MSU. Hemozoin appears to match the level of potency of these two activators. Unlike ATP that activates the Nalp3 inflammasome through P2X7 receptor-mediated K^+^ efflux, hemozoin requires crystal formation (the precursor heme is inactive) in the same way as MSU, where soluble uric acid does not activate the inflammasome. It is therefore not surprising that hemozoin and MSU use similar signalling pathways, including crystal phagocytosis, generation of ROS and K^+^ efflux.

The exact role which hemozoin plays in malaria pathogenesis is still a matter of debate. Although hemozoin was initially proposed to possess a direct neurotoxic role through activation of TLRs, murine cerebral malaria was recently shown to develop in the absence of TLR signalling [11], [12]. The obvious differences in these studies are not easily explained, as the experimental settings used are almost identical. Our results suggest that the Nalp3 inflammasome may also contribute to the effects of malaria and possibly also to the neurotoxicity. If future studies can confirm this notion, drugs that dampen hemozoin-triggered inflammasome activation may thus efficaciously complement conventional antimalarial drugs.

## Materials and Methods

### Mice

*Nalp3^−/−^* [21], *Asc^−/−^* and *Ipaf^−/−^* [23], *caspase-1^−/−^* [24], *MyD88^−/−^* [25], gp91phox^−/−^ (Jackson Laboratories, stock#002365) and *P2X7^−/−^* (Jackson Laboratories, stock#005576) mice (on C57BL/6J background) and *IL-1R^−/−^* [26] (on BALB/c background) were housed at the University of Lausanne following the Swiss Federal Veterinary Office guidelines. Six to ten week old C57Bl/6J-OlaHsd and BALB/c-OlaHsd mice were purchased from Harlan, The Netherlands.

### Reagents

Hemozoin was prepared as described [9]. Briefly, hemin chloride (from Sigma BioChemika, > 98% HPLC) was dissolved in degassed NaOH, the pH adjusted to 4.8 by addition of propionic acid and the solution left O/N at 70°C. The formed crystals were extensively washed in NaHCO_3_ several times, alternated with water rinses. Finally the crystals were washed with H_2_O and methanol alternatively, before being dried over phosphorus pentoxide. The crystals were then weighed and dissolved at a concentration of 10 mg/ml in PBS.

Nigericin, uric acid, cytochalasin D and z-VAD-fmk were purchased from Sigma and DPI from Alexis. Ultrapure LPS was obtained from Invivogen. Anti-human cleaved IL-1β (2021L) was purchased from Cell Signaling, and anti-IL1β p35 is a sheep antibody made in the Tschopp laboratory. The antibody against mouse IL-1β was a gift from Roberto Solari, Glaxo. The antibody against mouse caspase-1 (p20) was a generous gift from Dr. Peter Vandenabeele (Ghent University). Cytokine and chemokine detection was performed with the Cytometric Beads Array (CBA) kits from BD Biosciences. All tissue culture reagents were bought from Invitrogen.

### Generation of THP1 cells expressing shRNA

THP-1 stably expressing shNALP3, caspase-1, ASC and p22phox were obtained as previously described [27].

### Cell preparation

Bone-marrow macrophages were derived form tibia and femoral bone marrow cells as described elsewhere [28]. Mouse macrophages were primed overnight with 250 ng/ml ultra-pure LPS (Invivogen) and cell culture medium was removed before stimulation for 6 h in Optimem.

For experiments, THP-1 were differentiated 3 hours with 0.5 µM PMA. Cell extracts and precipitated supernatants were analyzed by western blot and CBA.

### *In vivo* mouse peritonitis model

Peritonitis was induced by injection of 0.25 mg of hemozoin or 0.2 mg zymosan in 0.5 ml sterile PBS. After 6 h, mice were killed by CO_2_ exposure and peritoneal cavities were washed with 6 ml of PBS. The lavage fluids were analysed for neutrophil influx by FACS (CD11b^+^ Ly-6C^+^ Ly-6G^high^ F4/80^−^). The following monoclonal antibodies were used: anti-CD11b (M1/70), anti-F4/80 (BM8) from ebioscience, anti-Ly-6C (AL-21) and anti-Ly-6G (1A8) from BD Biosciences. Samples were acquired on a FACSCanto (BD Biosciences) and analyzed by using the FLOWJO software (Tree Star).

### *Plasmodium berghei* ANKA infection

The *P. berghei* (ANKA strain) was maintained by alternating cyclic passage of the parasite in *Anopheles stephensi* mosquitoes and in BALB/c mice at the mosquito colony of the Department of Biochemistry, University of Lausanne. Sporozoites were collected by dissecting the mosquito salivary glands in DMEM 21 days after their infective blood meal. Each mouse was infected with 10 viable sporozoites by IV injection in the tail vein. After 5 days, infection of red blood cells (RBC) was monitored by microscopy of Giemsa-stained thin blood smears and used to calculate parasitemia (as % of infected RBC). 6–10 days after infection, susceptible mice started to develop symptoms of cerebral malaria (CM). Survival and signs of disease were monitored daily. Animals that showed neurological signs, such as convulsions, ataxia and paralysis, and died between 7 and 12 days after infection were considered to have CM. Brains were removed and used for histological analysis.

### Cerebral histopathology

Brains were fixed in buffered formol for 12 hours and paraffin-embedded. Cerebral tissue sections (2 µm) were stained with hematoxylin and eosin (HE) or Prussian blue using standard procedures. CD45 staining was performed after antigen retrieval with a TRIS/EDTA pH 9 solution. CD45/Ly5 antibody was from BD Pharmingen (#550539).

### Statistical analysis

Differences between groups were calculated using the unpaired t test (GraphPad Prism version 5.0). Differences were considered significant when p≤0.05.

## Supporting Information

Figure S1 (4.82 MB TIF)

Figure S2 (1.14 MB TIF)

Figure S3 (1.33 MB TIF)
