# Supplementary figures and images for: Malarial Hemozoin Is a Nalp3 Inflammasome Activating Danger Signal
Source: PLoS One. 2009 Aug 4;4(8):e6510. doi: 10.1371/journal.pone.0006510 (PMC2714977; doi:10.1371/journal.pone.0006510)

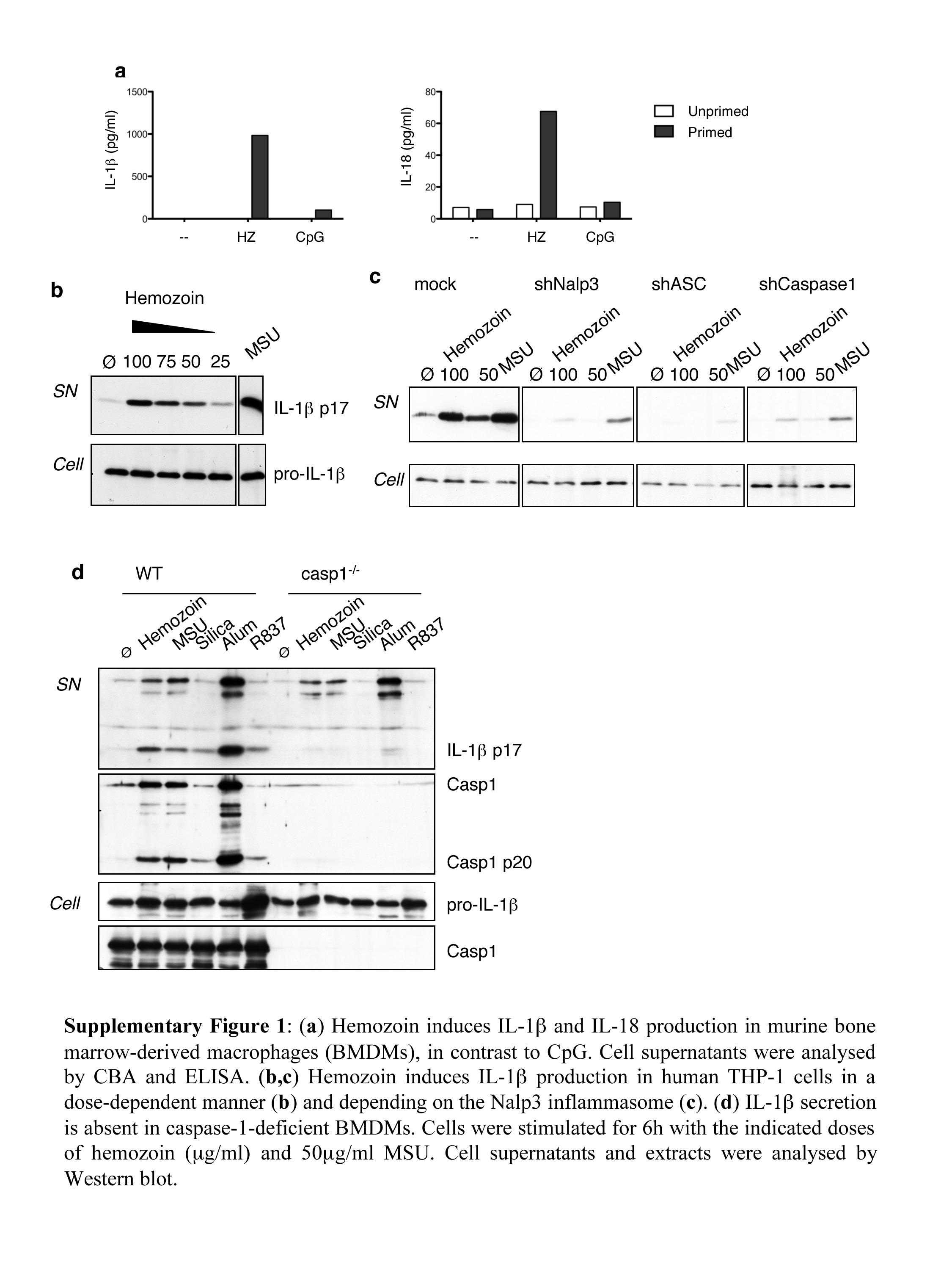

Supplement: Figure S1 — (4.82 MB TIF) [file pone.0006510.s001.tif]

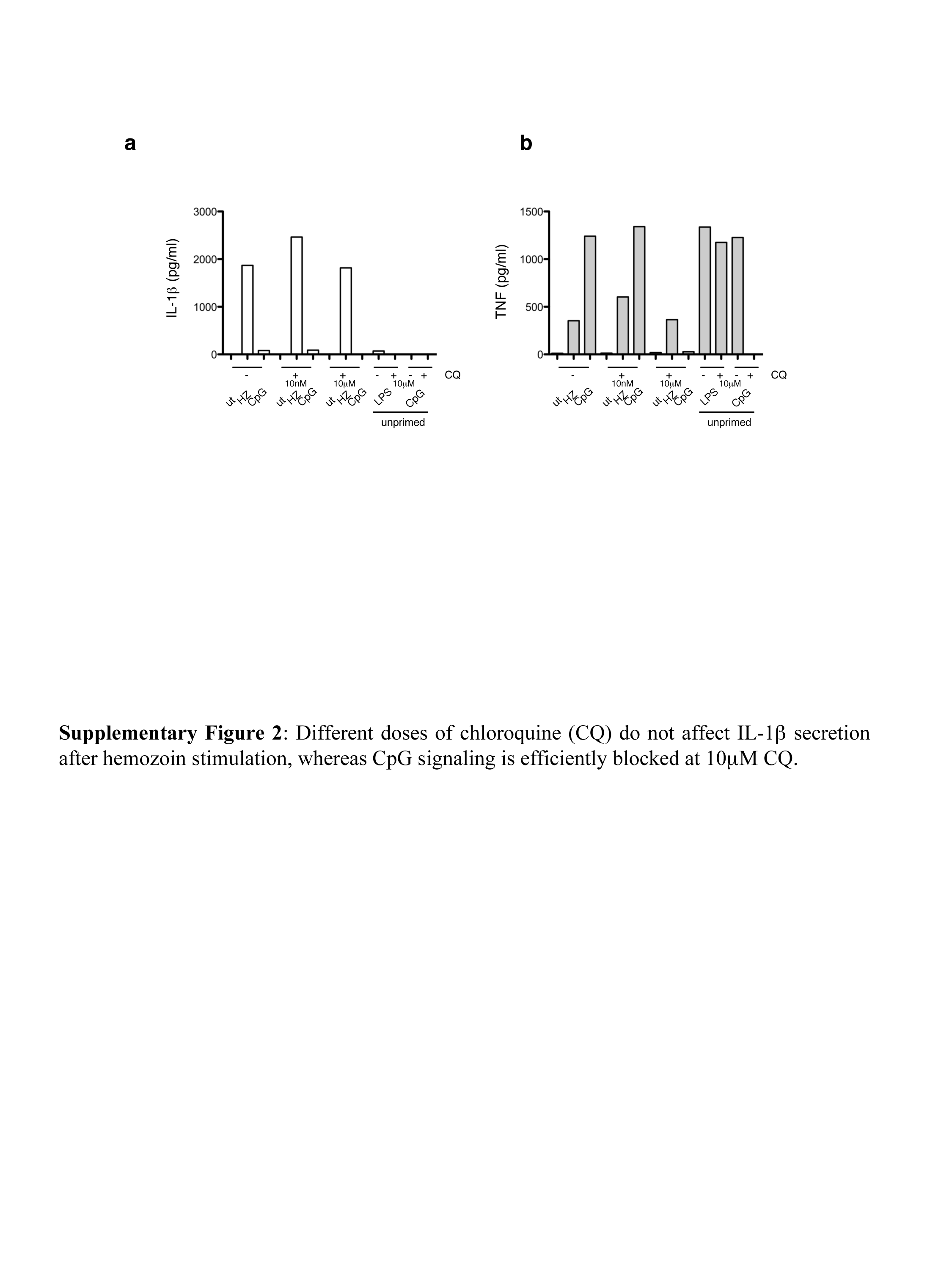

Supplement: Figure S2 — (1.14 MB TIF) [file pone.0006510.s002.tif]

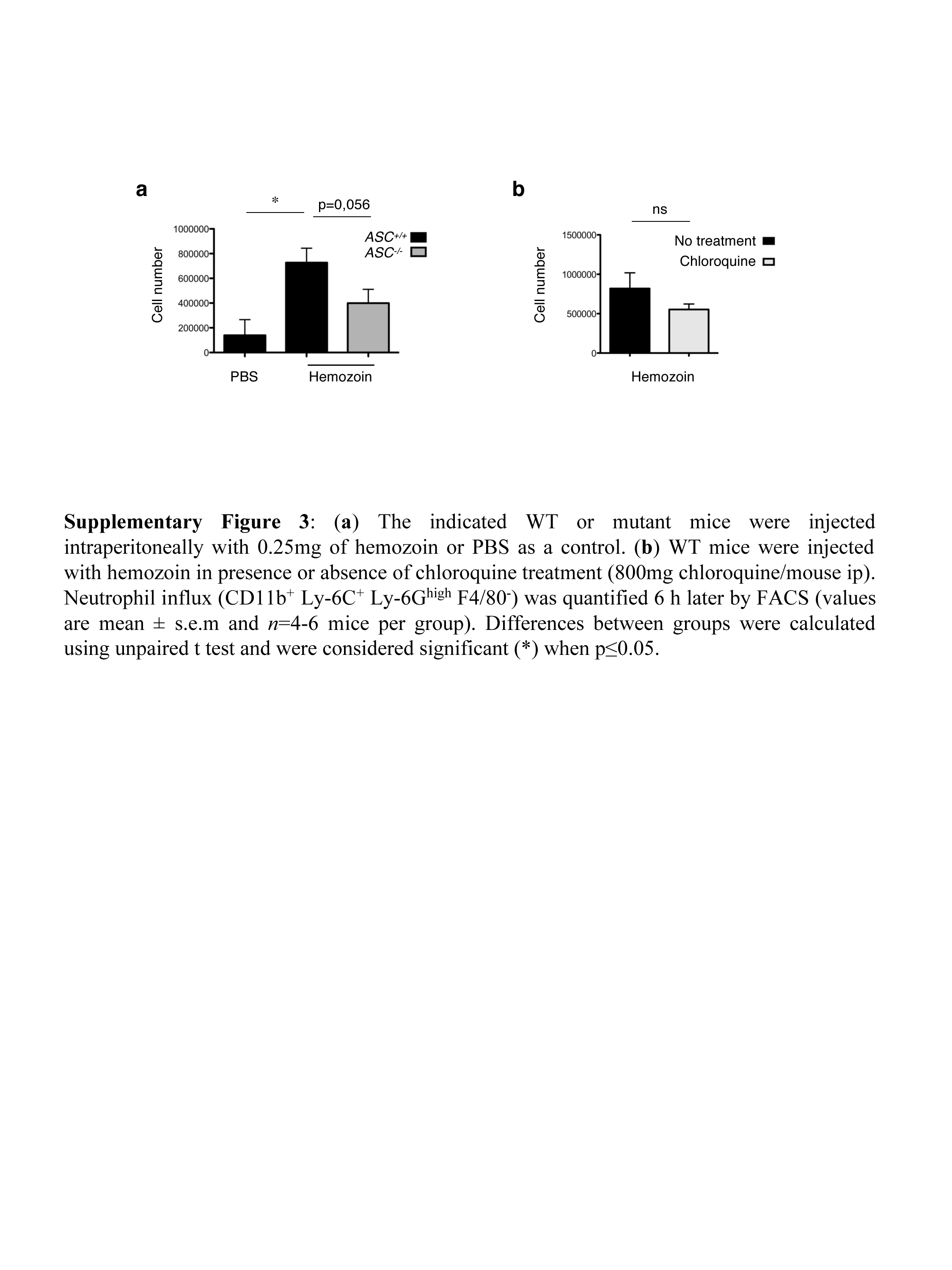

Supplement: Figure S3 — (1.33 MB TIF) [file pone.0006510.s003.tif]
